# Osseous Healing in Surgically Prepared Bone Defects Using Different Grafting Materials: An Experimental Study in Pigs

**DOI:** 10.3390/dj8010007

**Published:** 2020-01-09

**Authors:** Savvas Titsinides, Theodore Karatzas, Despoina Perrea, Efstathios Eleftheriadis, Leonidas Podaropoulos, Demos Kalyvas, Christos Katopodis, George Agrogiannis

**Affiliations:** 1 Dental School, University of Athens, 2 Thivon Street, 11527 Athens, Greece; 2 Laboratory of Experimental Surgery and Surgical Research “N. S. Christeas”, Medical School, University of Athens, 75 M. Assias Street, 11527 Athens, Greece; tkaratz@med.uoa.gr (T.K.); dperrea@med.uoa.gr (D.P.); 3 Department of Oral and Maxillofacial Surgery, Dental School, University of Athens, 2 Thivon Street, 11527 Athens, Greece; stathisele@gmail.com (E.E.); lpodar@otenet.gr (L.P.); demkal@dent.uoa.gr (D.K.); 4 Data Analysis, 7 Kozanis Street, 17673 Athens, Greece; christoskatopodis@outlook.com; 5 1st Department of Pathology, Medical School, University of Athens, 75 M. Assias Street, 11527 Athens, Greece; agrojohn@med.uoa.gr

**Keywords:** alveolar bone grafting, bone substitutes, bone regeneration, bone replacement material

## Abstract

Regeneration of large jaw bone defects still remains a clinical challenge. To avoid incomplete bone repair, bone grafts have been advocated to support the healing process. This study comparatively evaluated new bone formation among a synthetic graft substitute, a human bone derivative, and a bovine xenograft. Materials were placed in 3 out of the 4 bone cavities, while 1 deficit was left empty, serving as a control, in mono-cortical defects, surgically prepared in the porcine calvaria bone. Animals were randomized in 2 groups and euthanized at 8 and 12 weeks. Harvested tissue specimens were qualitatively evaluated by histology. New bone formation was quantitatively measured by histomorphometry. Maximum new bone formation was noticed in defects grafted with beta-tricalcium phosphate b-TCP compared to the other bone substitutes, at 8 and 12 weeks post-surgery. Bovine and human allograft induced less new bone formation compared to empty bone cavity. Histologic analysis revealed that b-TCP was absorbed and substituted significantly, while bovine and human allograft was maintained almost intact in close proximity with new bone. Based on our findings, higher new bone formation was detected in defects filled with b-TCP when compared to bovine and human graft substitutes.

## 1. Introduction

Regeneration of jaw bone deficits caused by accidents (traffic, occupational, sports, or shooting accidents), surgery (removal of benign lesions, malignant neoplasms, tooth extraction), congenital abnormalities (clefts or visceral skull bones hypoplasia), periodontal inflammation, and finally jaw atrophy (advanced age, systemic disease) still remains a clinical challenge [1].

To avoid the unfavorable outcome of incomplete bone repair, bone grafts have been advocated to support the healing process.

In general, the ideal grafting material should ultimately be fully replaced by high quality host bone, exhibiting synchronized degradation rate in relation to new bone development for complete regeneration [2].

Bone graft substitutes of diverse origin and composition possess different bone regenerative potential associated with the following properties: (1) Osteogenesis: living osteoblasts derived from the graft contribute to the production of new bone; (2) Osteoinduction: stimulation of osteoprogenitor cells that differentiate into osteoblasts, usually influenced by a bone morphogenetic protein released from the graft; (3) Osteoconduction: graft structure that provides a scaffold, aiding capillaries and precursor bone cells to develop [3].

Among these materials, autogenous bone is considered the ‘gold standard’, presenting osteoconductive, osteoinductive, and osteogenetic properties. This last feature, in particular, is unique to autografts since they contain vital osteoblasts, supporting osteogenesis [4]. Other benefits within this category are exclusion of disease transmission and allergic reactions while drawbacks include restricted availability, the need for an additional surgical donor site, increased morbidity, and extended operating time [5].

As an alternative, other bone grafts types have been developed, including allografts, xenografts, and alloplasts, possessing different properties according to their embryologic origin, histologic architecture, structure, and physiology of graft resorption—new bone formation [6].

Alloplastic bone substitutes are available in many sizes, shapes, and unlimited quantity, combined with elimination of transmitting infections. Cutting-edge technology is employed to improve surface texture, mineral formation, and biocompatibility, so that the final product may mimic, to a great extent, the osseous natural environment. These biomimetic materials are characterized by osteoconductive and, in some cases, osteoinductive potential on their own [7,8]. One of the most popular materials of this category is ceramic beta-tricalcium phosphate (b-TCP), featuring compressive strength similar to that of cancellous bone while undergoing resorption by hydrolysis, enzymatic and phagocytic processes [9].

In the xenografts category, bone substitutes of bovine origin that are deproteinized and lyophilized were the first to be applied to patients. They are commercially available in a wide range of products and considered among the most documented materials of this class. They have osteoconductive properties, causing no immune response, however, they are subjected to poor or slow absorption due to high temperature processing, surrounded by newly formed bone rather than entering the normal bone remodeling process [3,10].

Allografts derived from humans, either living donors—usually during femoral head replacement—or cadaveric bone material, are selected and properly processed to neutralize the immune response and transmission of infectious diseases, being finally preserved in bone banks. These bone grafting products are characterized by osteoconductive and osteoinductive properties [6].

While no species fulfills all requirements of an ideal experimental prototype, reflecting only approximations to human physiology, the pig as an animal model exhibits a new bone formation rate of 1.2–1.5 μm/day, similar to that described in humans [11]. Other features favoring this animal are its similarity to human organism as to the healing process, bone remodeling, mineral concentration, and lamellar structure, with pig calvaria represents a suitable region for interpretation of results to the jaws, given its embryologic and morphologic similarity to the maxillary region, its relatively poor blood supply, and satisfactory bone quantity. Few drawbacks described for this species include large growth rates, excessive final body weight, difficulty in handling, and a relatively noisy and aggressive attitude [12].

Although, in most studies, a bi-cortical (full thickness) defect is used, disadvantages regarding skull bones include destruction of the dura mater and sagittal sinus, dislocation of the bone substitute, and fibrous healing on both sides of the deficit, preventing osseous regeneration [13,14]. On the other hand, the mono-cortical defect in the porcine calvaria involves a procedure that provides information in a less complicated manner as to the influence any investigated filling material on bone regeneration [15].

The purpose of this study was to evaluate in vivo development of newly formed bone using 3 commercially available bone grafting materials in mono-cortical defects in the calvaria bone of pigs. In detail, a synthetic graft substitute, a human bone derivative, a bovine xenograft, and an empty bone cavity left to follow natural bone healing were all compared, both qualitatively and quantitatively, in surgically prepared osseous cavities using histologic and histomorphometric analysis.

## 2. Materials and Methods

### 2.1. Animals

This protocol meets the guidelines and rules for research on animals as stated by the Institutional Animal Care and Use Committee of the Veterinary Department, Greek Ministry of Rural Development and Veterinary, Attica Prefecture, Greece (1265/11-05-2015).

Sixteen adult male pigs (Landrace/Large White) were included in the study, acclimatized under the same environmental conditions for a period of 7 days before initiation of the experiment and fed with a balanced diet in individual cages to “N. S. Christeas”, Laboratory of Experimental Surgery and Surgical Research, Medical School, University of Athens, Greece.

### 2.2. Surgical Procedure and Experimental Design

For induction of anesthesia, ketamine hydrochloride (Imalgene, Merial, Lyon, France) at a dosage of 25 mg/kg body weight combined with intramuscular injection of 2 mg/kg body weight xylazine (Rompun, Bayer Hellas AG, Athens, Greece) was applied. General anesthesia by orotracheal intubation was maintained with iv propofol (Diprivan, AstraZeneca, London, UK).

At the surgical field of interest, disinfection of the skin of the skull was achieved using povidone iodine (Betadine, Lavipharm, Athens, Greece) solution. Topical infiltration of local anesthesia with 4% articaine hydrochloride combined with 1:100,000 epinephrine (Ubistesin Forte, 3M ESPE, Athens, Greece) was also performed to induce hemostasis. Throughout the procedure, the animals were provided 100% oxygen and monitored for vital signs.

**Figure 1:**
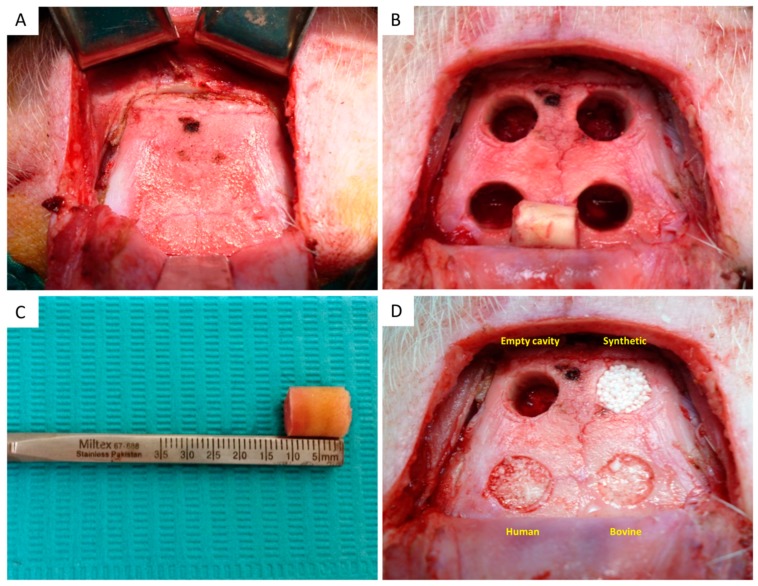
(**A**) Surgical exposure of the calvaria bone after elevation of a full thickness flap, (**B**) Intra-operative view of the surgically prepared bone defects, (**C**) The cylindrical bone segments were gradually mobilized and detached using a thin chisel, (**D**) Bone defects filled with graft substitutes while one was left empty.

A semicircular incision was made in the skin over the top of the cranial vault, with subsequent elevation of a cutaneous full-thickness flap, exposing the calvaria bone (Figure 1A). During the next step, 4 mono-cortical defects (10 mm depth/10 mm diameter) of cylindrical shape were performed—as previously described by Schlegel et al.—using a trephine bur (Meisinger, Neuss, Germany) with rotation speed of 250 rpm and flushing of 0.9% physiologic saline, with a 10 mm distance between them to avoid possible biologic interactions (Figure 1B) [15]. The cylindrical bone segments were gradually mobilized and detached using a thin chisel (Figure 1C). All sites were thoroughly debrided from soft tissues and rinsed with sterile saline. Bone grafts of synthetic (Calc-i-oss classic, Sunstar Guidor, Switzerland) bovine (Bio-Oss, Geistlich Pharma AG, Wolhusen, Switzerland) and human origin (Demokritos, National Centre for Scientific Research tissue bank, Athens, Greece) were placed in 3 out of the 4 defects, while 1 defect was left to heal without graft implantation, serving as a control (Table 1, Figure 1D). Wound closure was successfully accomplished using 3/0 silk sutures (Ethicon, Johnson & Johnson, Somerville, NJ, USA), followed by povidone iodine (Betadine, Lavipharm, Athens, Greece) solution, applied on the skin.

Animals were monitored for 2 h in the recovery room, further given appropriate intramuscular antibiotic prophylaxis with cefuroxime 750 mg (Zinacef, Glaxosmithkline, Athens, Greece) as well as an analgesic suppository of paracetamol (Depon, Bristol-Myers Squibb, Athens, Greece).

### 2.3. Sacrifice of Animals

The animals were randomly divided into 2 groups (8 animals per group) and sacrificed with an intravenous injection of sodium thiopental (Pentothal, Abbott Hellas, Athens, Greece) on the 8th and 12th post-operative week, respectively, to evaluate contribution of bone grafts to bone regeneration.

This was followed by the use of a mechanical saw to remove the skull bones containing the bone defects that were further cut into 4 mm-thick frontal sections. The 2 most representative sections from the central part of the deficits were selected for each bone defect and were embedded in tissue cassettes while the samples were further processed.

### 2.4. Histologic Analysis

The block specimens were fixed in 10% formalin for 2 days and subsequently decalcified in bone decalcification solution (Diapath S.p.a., Martinengo, Italy) for 21 days with a liquid change every 7 days. Later on, tissue specimens were embedded in paraffin and 3 µm-thick sections were prepared and stained with hematoxylin and eosin, properly examined under a light microscope (Nikon Eclipse 80, Nikon, Tokyo, Japan) at a minimum ×40 magnification. The entire section was evaluated for macroscopic parameters only qualitatively.

### 2.5. Histomorphometric Analysis

Examinations were performed under a light microscope (Nikon Eclipse 80, Nikon, Tokyo, Japan) at a magnification ×40. From every pair of selected bone section, the most appropriate was selected and this area was acquired with a digital camera microscope unit (Nikon DS-2MW, Nikon, Tokyo, Japan). The sections were merged and processed using image analysis software (Image-Pro Plus v. 5.1, Media Cybernetics, Rockville, MD, USA) and the appropriate mask was created for the area of newly formed bone.

Histomorphometric evaluations consisted of measurements regarding the percentage of new-formed bone to the total volume of the site of interest (BV/TV) (bone volume/tissue volume).

### 2.6. Statistical Analysis

Pairwise comparisons were performed using the Bonferroni test. In case of non-normal data distribution, the Mann–Whitney test was used. Data were expressed as mean ± standard deviation. All tests were two-sided, and statistical significance was set at *p* < 0.05.

## 3. Results

### 3.1. Overall

Surgical intervention was uneventful and during post-operative course till sacrifice, 15 animals exhibited no complication except 1 that died before completion of the 12 weeks healing period. Also, at the time points of euthanasia, no signs of local inflammation–infection around the skin of the skull were detected.

**Figure 2:**
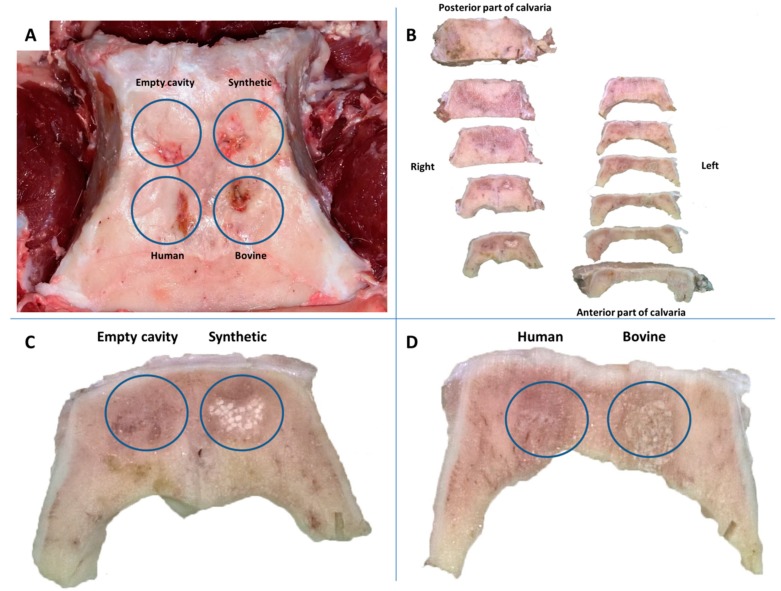
(**A**) Gross view of the bone defects after sacrifice of animals. All sites were filled with new bone, (**B**) Frontal sections of the calvaria bone during processing, (**C**,**D**) Frontal sections in magnification. Notice the remnants of the grafts while in the empty cavity soft tissue can be identified.

After debridement of the soft tissues, it was interesting that the areas of the bone defects appeared macroscopically similar, fulfilled with bone, exhibiting only few residual deficits. However, after frontal gross sectioning of the calvaria bone, it was noticed that at areas implanted with the grafting products, residual particles could be identified to a greater or lesser extent. As to empty cavities, it was observed that a part of the defect was fulfilled with a relatively soft tissue (Figure 2).

### 3.2. Histology

Light microscopy allowed qualitative analysis as to bone healing and regeneration process within the defects. Comments are listed below:

A. 8 weeks

At 8 weeks post-surgery, histologic analysis exhibited presence of new bone tissue in all cavities filled with bone substitutes, woven and partially lamellar, while no signs of inflammatory response, necrosis, or foreign body reaction were observed. The graft particles were surrounded by or were in contact with lamellar bone, demonstrating good osteoconductivity and biocompatibility.

**Figure 3:**
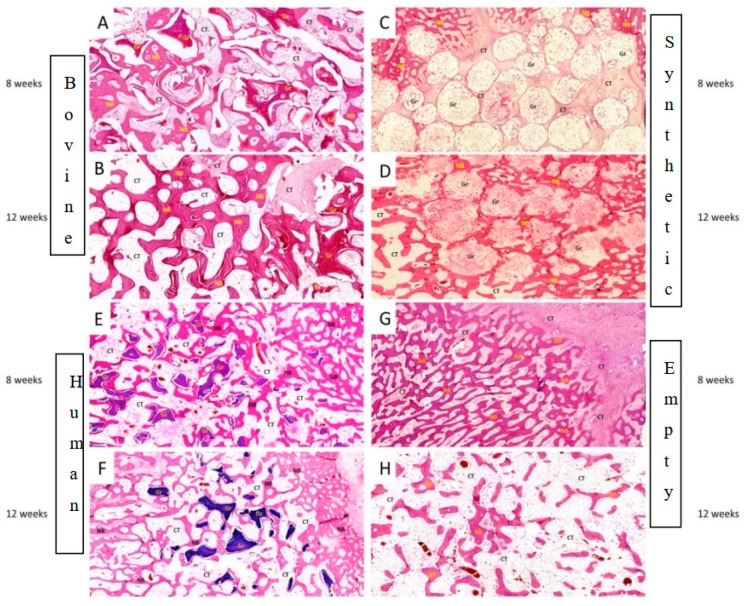
Representative histological pictures at 8 and 12 weeks of bone healing (H&E staining) (original magnification ×40). Residual granules of the grafting materials are still apparent, (**A**,**B**) Bovine graft, (**C**,**D**) Synthetic graft, (**E**,**F**) Human graft, (**G**,**H**) Empty bone cavity gradually forming new bone intermixed with connective tissue. Abbreviations: NB: new bone, Gr: grafting material, CT: connective tissue.

Spaces between the graft particles and the newly formed bone were characterized by copious mesenchymal cells and vessels while osteoblasts and multi-nucleated osteoclasts could be identified on the periphery of the grafts surface. Presence of residual graft was observed in the surgical defects filled with alloplastic material as well as the bovine and human graft. In particular, granules of bovine graft appeared stable, almost intact, without resorption lacunae in the context of the absorption process. In the empty deficits, small amounts of new bone tissue were formed at the margins of the defect with a tendency of extension towards the central part where the greater proportion was filled with a layer of fibrous connective tissue (Figure 3A,C,E,G).

B. 12 weeks

At 12 weeks, more pronounced bone formation was noted, with lamellar bone being more prominent, surrounding all graft types, whereas, fibrous connective tissue was found to be less compared to mesenchymal tissue observed at 8 weeks.

It was obvious that b-TCP granules were deconstructed to a sufficient degree, with signs of both dissolution of the b-TCP particles and direct cellular resorption. These granules were predominantly embedded in the newly formed bone which was more interconnected than at 8 weeks, with signs of bone remodeling. In addition, the bovine and human allograft exhibited osseous integration with mature bone, to a greater extent, compared to 8 weeks, though particles seemed relatively intact, almost without evidence of absorption and bone substitution. In non-grafted cavities, bone maturation had increased significantly, mainly in the periphery of the deficits (Figure 3B,D,F,H).

### 3.3. Histomorphometry

Results of histomorphometric analysis are shown in Table 2. Based on these findings, new bone formation was noticed, to a greater extent, in defects grafted with b-TCP compared to other substitutes in 8 and 12 weeks. An interesting finding was that while b-TCP exhibited less new bone compared to empty cavity at 8 weeks, this material surpassed unfilled defect bone formation at 12 weeks. Less bone formation volume was noticed in bovine and human allograft compared to empty cavity, in 8 and 12 weeks.

## 4. Discussion

During this project, histomorphometrιc evaluation revealed that bone defects filled with b-TCP were characterized by a more pronounced bone formation compared to other grafts in both 8 and 12 weeks post-surgery. These results seem to be in agreement with many other research protocols that described optimum bone regeneration in alloplastic bone substitutes containing b-TCP. A recent study measuring newly formed bone volume in intact extraction sockets fulfilled with a resorbable alloplastic biomaterial, consisting of b-TCP and polylactide-co-glycolide, concluded that osseous regeneration was more pronounced compared to the empty unassisted sites where natural healing occurred. This finding, however, was not statistically significant, possibly, according to authors, due to the minimum number of animals used and presence of factors such as maintenance of buccal bone plate after extraction and flapless surgery [16]. Also, another experiment using micro computed tomography-CT described that the highest vital bone content was noticed in defects grafted with b-TCP and calcium sulfate (b-TCP/CS), followed by sockets with no graft material and a bovine xenograft in rabbit calvaria defects, 8 weeks after implantation [17]. The objective of another recent article was to evaluate an in situ hardening biphasic hydroxyapatite/b-TCP bone graft substitute for ridge preservation after tooth extraction without primary wound closure or a barrier membrane. Histomorphometric analysis of core biopsy samples showed that using this substitute results in an effective preservation of the ridge contour and sufficient new bone formation [18]. A systematic review analyzed the outcomes of randomized controlled trials of socket grafting procedures performed with flapless extraction of teeth after a minimum healing period of 12 week. The mean histologic outcomes at or beyond the 12-week re-entry period revealed the highest vital bone content for sockets grafted with alloplasts, followed by sockets with no graft material, xenografts, and allografts [19]. According to another publication, b-TCP/CS implanted in cranial defects in rabbits resulted in 26.28% and 38.47% median values for new bone volume at 3 and 6 weeks, characterized by the authors as a biocompatible and osteoconductive material [20]. Yang et al., using micro-CT analysis to study the performance of a b-TCP/CS bone substitute compared to available polymethylmethacrylate cement and empty cavity in a sheep vertebral bone defect model, reported a higher new bone development of b-TCP/CS at 8, 16, and 36 weeks of observation [21]. In vivo histologic evaluations estimating the osteogenic potential of b-TCP/CS and human demineralized bone matrix putty in the mandible of rabbits confirmed that both grafts possess osteogenic activity and can support new bone formation, although at a slower rate than the spontaneous healing response [22]. Podaropoulos et al., evaluating with histomorphometric analysis b-TCP/CS in comparison with pure b-TCP in surgically prepared bone defects on the iliac crest of Beagle dogs, reported that b-TCP/CS produced 49.38% new bone compared to 40.31% using b-TCP alone and 17.77% on empty cavity, 4 months after implantation [23]. Our histologic findings confirm earlier animal studies documenting adequate resorption of b-TCP, while decrease of granules seems to be a combination of dissolution and direct cell-mediated resorption [24]. One possible hypothesis for relatively fast absorption of this bone substitute is its chemical composition, with a Ca/P ratio of 1.5, as well as its porous ceramic structure [3].

The fact that 2 observation periods were used in this study enabled the opportunity to identify changes in bone formation potential at the tissue level.

Indeed, an interesting finding of our study was that while at 8 weeks, b-TCP produced less new bone compared with empty cavity, at 12 weeks this grafting material was found to surpass empty cavity as to bone formation. Based on the results of our study, Hong et al. reported that the production of new bone in post-extraction sockets in dogs where b-TCP was inserted was more pronounced in the advanced maturation phase of bone healing at 8 weeks, while this material exhibited low bone formation at 2 and 4 weeks [24]. A relative analogous result is also described in a study where b-TCP exhibited delay compared to autograft in the early stages, but at 8 weeks presented more pronounced bone production [25]. These results, although initially raising questions about the efficacy of grafting materials, can be answered by considering the potential changes that these materials may have in the healing process. As already reported in previous studies, cellular growth processes with the addition of grafts may complicate, inhibit, or delay bone healing [26,27]. At this point, it is worth noting that at the molecular level, inflammatory cytokines such as IL-1b and TNFa may impair bone regeneration by inhibiting osteoblast differentiation and maturation as well as inducing osteoclast activity [28,29]. Trying to interpret results in our analysis, it seems that while inflammatory absorptive process for b-TCP is more acute at 8 weeks, it begins to cease during the maturation phase of bone healing at 12 weeks, allowing for more intense new bone formation.

As to bovine and human allograft used in our project, new bone formation was found to be less compared to b-TCP, possibly due to the slow resorption rate of the formers. These results reconfirm previous findings that long-term presence of residual non-resorbable or slowly resorbable particles of the graft might deregulate bone healing and the remodeling mechanism depending on the biodegradation pattern, having negative results on the overall quality, quantity, and architecture of the reconstructed bone [30]. These opinions raise dilemmas as to whether bovine graft is actually resorbable, an issue requiring further research. In line with this possibility, on our histological sections in the bovine graft, osteoclast-like multinucleated cells were identified in close approximation to graft granules, however, with absence of resorption lacunae. Multinucleated giant cells were also observed in relation to b-TCP, but, in contrast to the bovine graft, they were not arranged along the b-TCP remnants, rather being actively phagocytizing b-TCP particles, as described in previous studies [31]. Based on these findings, Artzi et al. found total resorption of b-TCP particles compared with inorganic bovine bone 24 months post-surgery in a canine model [32]. Mordenfeld et al., performing histologic and histomorphometric analysis of bone harvesting 11 years after sinus floor augmentation with deproteinized bovine and autogenous bone, stated that the xenograft particles were not resorbed but were well-integrated in lamellar bone, with no significant changes in particle size [33]. According to a recent study, evaluating bovine graft absorption after sinus elevation in rabbits, 8 weeks post-surgery, reported that this material is resorbed very slowly, leading to low new bone formation since biodegradation is an important factor for bone regeneration [34]. A relatively conflicting result in studies with bovine bone grafts is the fact that although new bone formation is not the maximum in this type compared to other grafts, total fractions of mineralized tissue (graft and bone) is sometimes found to be higher in relation to other grafts. One explanation might be that complete incorporation of the bovine graft particles within new bone, is leading to a dense, hard tissue network overall. This intermixed, dense, hard tissue element was also identified in our histologic sections, where osseous development was in direct contact with bovine graft particles, exhibiting lack of inflammation or connective tissue development. With respect to the human allograft as already reported, decreased bone regeneration and absorption rate of the fragments was observed in our analysis compared to b-TCP. A potential mechanism for these phenomena is the increased immune response that could inhibit bone regeneration [35]. According to another possible explanation, freeze-dried mineralized human allografts, like in our project, exhibit a slow rate of biodegradation and remain trapped within the surrounding newly formed bone, while after mineralization, resorption capability is suspended [36,37].

Based on our histologic observations, it is possible to argue that graft replacement and bone regeneration follow a different pattern in each of the grafting material examined at tissue level, a comment described by previous studies as well [26,38].

Though larger new bone production observed in our study within the empty cavity, compared to all bone grafts at 8 weeks and bovine and human graft substitute at 12 weeks, seems initially puzzling, it can be explained by the theory that the relatively reduced absorptive process of these materials delays the production of new bone. Drawbacks including the weakness of the empty cavity to support the structural integrity of bone deficit from soft tissue penetrance as well as incomplete fulfillment of the deficit but only in the periphery since growth factors are relatively less in concentration in such defects reinforce the use of grafting materials even if they exhibit some delay regarding bone regeneration.

The present study compared the osteogenic potential of bone grafts of synthetic, bovine, and human origin as well as natural bone healing of an empty cavity, in a previously established pig model by qualitatively and quantitatively analyzing new bone formation using histologic and histomorphometric analysis.

The results of this project are listed below: (1) b-TCP graft exhibited more pronounced new bone formation compared to bovine and human graft substitutes at 8 and 12 weeks; (2) While b-TCP induced less bone compared to empty cavity at 8 weeks, its bone production overcame empty defect during bone healing maturation at 12 weeks; (3) Bovine and human materials developed less bone in comparison to the control at 8 and 12 weeks; (4) According to histologic analysis, b-TCP was found to be characterized by the most significant rate of resorption between 8 and 12 weeks, compared to human allograft and bovine xenograft, where particles were spotted to be among these time moments almost intact, without signs of significant absorption ability. It seems that bone regeneration among these grafting materials follows different patterns.

## Figures and Tables

**Table 1:** Grafting materials applied in our study and their characteristics.

Graft	Characteristics	Granule Size (mm)
Bio-Oss	Bovine, deproteinized bone mineral	1–2
Calc-i-oss	Synthetic, phase-pure beta-tricalcium phosphate	1–1.6
Demokritos	Human, freeze-dried, mineralized, and lyophilized cancellous bone allograft	1–2

**Table 2:** Results of histomorphometric analysis regarding total volumes (%) of newly formed bone in the empty, synthetic, bovine, and human grafted sites (mean). Measurements were considered statistically significant at *p* < 0.05.

	**8 Weeks**		**12 Weeks**	
**Group**	**Mean**	**SD**	**Mean**	**SD**
Control	34.81	7.98	44.80	0.60
Synthetic	34.11	20.21	47.25	1.12
Bovine	32.08	11.95	39.14	1.10
Human	29.20	9.55	35.08	0.93
**Group comparison**	***p* value**			
	**8 weeks**		**12 weeks**	
Control Synthetic	0.858		0.005	
Control Human	0.179		0.001	
Control Bovine	0.541		0.001	
Synthetic Bovine	0.693		0.002	
Synthetic Human	0.315		0.001	
Bovine Human	0.583		0.001	
